# FKBP11 improves the malignant property of osteosarcoma cells and acts as a prognostic factor of osteosarcoma

**DOI:** 10.18632/aging.204523

**Published:** 2023-04-01

**Authors:** Duo Zeng, Jiayu Li, Xuhui Yuan, Feng Cai, Bo Yu, Lang Liu, Qinchan Chen, FeiFei Zhang, Yiping Liang, Xiaofeng Tang, Yuanxiang Peng, Gaoyang Qu, Pengyun Wu, QuanHui Jiao, Longhua Sun, Xiao-Bin Lv, Qi Liao

**Affiliations:** 1 Jiangxi Key Laboratory of Cancer Metastasis and Precision Treatment, Central Laboratory, The First Hospital of Nanchang, The Third Affiliated Hospital of Nanchang University, Nanchang, Jiangxi 330008, P.R. China; 2 Department of Orthopedics, The First Hospital of Nanchang, The Third Affiliated Hospital of Nanchang University, Nanchang, Jiangxi 330008, P.R. China; 3 College of Pharmacy, Jiangxi University of Chinese Medicine, Nanchang, Jiangxi 330004, P.R. China; 4 Departments of Pulmonary and Critical Care Medicine, The First Affiliated Hospital of Nanchang University, Nanchang, Jiangxi 330006, P.R. China

**Keywords:** osteosarcoma, FKBP11, MAPK signaling pathway, prognosis

## Abstract

Background: Osteosarcoma has become the most common bone malignancy in adolescents. Although the clinical treatment of osteosarcoma has advanced considerably in recent years, the 5-year survival rate has not improved significantly. Recently, many studies have shown that mRNA has unique advantages as a target for drug therapy. Therefore, this study aimed to identify a new prognostic factor and provide a new target for the treatment of osteosarcoma to improve the prognosis of patients.

Methods and results: We selected prognostic genes that are closely associated with osteosarcoma clinical features by obtaining osteosarcoma patient information from the GTEx and TARGET databases, and then we developed a risk model. We detected the expression of FKBP11 in osteosarcoma by qRT-PCR, western blotting, and immunohistochemistry and performed CCK-8, Transwell, colony formation, and flow cytometry assays to reveal the regulatory role of FKBP11. We found that FKBP11 was highly expressed in osteosarcoma; silencing FKBP11 expression suppressed the invasion and migration of osteosarcoma cells, slowed cell proliferation, and promoted apoptosis. We also found that silencing the expression of FKBP11 led to inhibition of MEK/ERK phosphorylation.

Conclusions: In conclusion, we validated that the prognostic factor FKBP11 is closely associated with osteosarcoma. Additionally, we identified a novel mechanism by which FKBP11 ameliorates the malignant properties of osteosarcoma cells through the MAPK pathway and serves as a prognostic factor in osteosarcoma. This study provides a new method for the treatment of osteosarcoma.

## INTRODUCTION

Osteosarcoma is highly malignant and is common in adolescents. It mainly arises from mesenchymal tissue [1] but is rarely found in soft tissues. It has a high incidence in the diaphysis. In recent decades, the 5-year survival rates for patients with osteosarcoma have improved to 70-80% with extensive resection and multidrug adjuvant chemotherapy [2, 3]. However, patients who undergo chemotherapy have low sensitivity to second-line chemotherapeutic agents, and their 5-year survival rate is significantly reduced to 20%. Therefore, it is essential to identify an effective prognostic factor to provide a new therapeutic target for osteosarcoma to improve the prognostic outcome of osteosarcoma patients. Recently, several studies have shown that many genes have been used as targets for drug therapy and that they offer unique advantages [4]. Furthermore, there is growing evidence that the mRNA levels of certain crucial cancer-related genes may serve as useful biomarkers for the diagnosis and prognosis evaluation of a variety of cancers, including breast cancer [5], clear renal cell carcinoma [6], and mucinous epidermoid carcinoma [7].

Bioinformatics is a very popular way to analyze biological data by searching (collecting and filtering) data, processing (editing, organizing, managing, and displaying) data, and utilizing (calculating and simulating) data through available tools. Bioinformatics analysis has an important role in exploring diagnostic and prognostic biomarkers for osteosarcoma. A study by Y. Liu et al. [8]. Identified that S100A9 could be a biomarker for osteosarcoma by bioinformatics analysis; further research showed that this biomarker could also promote the proliferation and invasiveness of osteosarcoma cells. In addition, WNT6 was found to be an indicator for the diagnosis and prognosis evaluation of osteosarcoma [9]. The level of MT2A has been identified as a biomarker for predicting chemoresistance, thereby providing a rational preclinical basis for applying MT2A-targeted therapy as a method to enhance the innate response to antitumor chemotherapy [10]. Despite these findings, our understanding of the prognostic role of mRNA levels in osteosarcoma is not completely clear, so it is still necessary to identify more biomarkers for osteosarcoma diagnosis and prognosis prediction. By analyzing the expression profiles and corresponding clinical data for 396 normal bone tissues from the Genotype-Tissue Expression (GTEx) database and 88 osteosarcoma samples from the Therapeutically Applicable Research to Generate Effective Treatments (TARGET) database, we constructed a risk assessment model based on FKBP11 and BNIP3 expression that can classify osteosarcoma patients into high and low risk groups and accurately predict the prognosis of patients, thus guiding the clinical treatment of osteosarcoma patients. In addition, we showed that STC2 was highly expressed in osteosarcoma tissues compared to normal tissues. Furthermore, FKBP11 was found to play a cancer-promoting role in osteosarcoma by vitro assays. Our findings revealed the value of FKBP11 in predicting osteosarcoma patient prognosis. These results provide a new target for the treatment of osteosarcoma in the future.

## MATERIALS AND METHODS

### Data acquisition

Information on 396 normal bone tissue samples was obtained through the GTEx database, and 88 osteosarcoma samples were downloaded from the TARGET database. The clinical information corresponding to these samples was also obtained from the platform.

### Enrichment analysis

R language was used to perform gene ontology (GO, http://www.geneontology.org) and Kyoto Encyclopedia of Genes and Genomes (KEGG) pathway enrichment analysis to find tumor prognosis-related genes and to perform functional annotation of core genes. These genes were screened by the online enrichment analysis platform Enrichr and the Molecular Signatures Database (MSigDB). *P* < 0.05 indicated a significant difference.

### Construction of the prognostic gene-based risk assessment model

The limma package and the “heatmap” package were applied to identify the differentially expressed genes between normal samples and osteosarcoma samples and create heatmaps, respectively. We performed univariate Cox regression analysis and least absolute shrinkage and selection operator (LASSO) Cox regression analysis on these genes; the genes with *P* < 0.05 in the univariate analysis, which were predicted to be related to the prognosis of osteosarcoma, were subjected to multivariate Cox analysis. After these analyses, a risk score model was obtained. The risk score was calculated as follows.


$$\text{Risk}\;\text{score}\;=\;\sum_{i\;=\;1}^{n}\;{\text{Coef}}_{i}\times \;x_{i}$$

where Coef is the coefficient and *x* is the expression level of each selected gene. Based on the median risk score, the patients with osteosarcoma were divided into low-risk and high-risk groups, and survival curves were plotted.

### Cell culture

Frozen osteosarcoma cells (MG63, 143B, U2R, U2OS) were removed from liquid nitrogen, thawed, revived and cultured routinely. The culture medium was DMEM containing 10% fetal bovine serum (FBS), and the cells were grown in an incubator with saturated humidity, a temperature of 37°C, and 5% CO_2_. The normal osteoblast cell line HFOB1.19 was cultured in DMEM-F12 medium (10% FBS, 0.3 mg/ml G418, 1% penicillin, and streptomycin) at 33.5°C and 5% CO_2_. The cells were split every two days.

### Quantitative real-time PCR assay

The cells to be treated were collected, total cellular RNA was extracted according to the TRIzol instructions (Vazyme Biotech Co., Ltd.), and quantitative real-time PCR (qRT-PCR) was performed. Reverse transcription was performed according to the instructions for the PrimeScript™ RT kit (TaKaRa China), and qRT-PCR was performed by using SYBR. The primer sequences for qRT-PCR in this paper was follows.

**Table:** 

FKBP11:	forward: 5′-ACACGCTCCACATACACTACACGG-3′
	reverse: 5′-ATGACTGCTCTTCGCTTCTCTCCC-3′ [11]
GADPH:	forward: 5′-GCCACCGTCAAGGCTGAGA-3′
	reverse: 5′-TGGTGAAGGGAACGCCAGT-3′

### Transwell assay and colony formation assay

Stromal gel was diluted with a prechilled serum-free medium at a ratio of 15:1 and added to chambers in a 24-well plate. Then, chambers containing stromal gel (for invasion assay) were placed in an incubator at 37°C for 2 hours. Then, 500 μl of serum was added to the lower chamber of the 24-well plate, and the cells were resuspended in serum-free medium. The cells were inoculated at a density of 1 × 10^5^/well into chambers containing stromal gel (for invasion assays) or without stromal gel (for migration assays) and incubated at 37°C for 24 hours. After incubation, the chambers were fixed with formaldehyde, placed in crystal violet solution for staining and then washed with PBS. Five randomly selected fields of view were observed and counted under a high-magnification microscope. The above experiments were repeated three times. For colony formation, after digestion and transfection with Si-FKBP11, the cells were collected in centrifuge tubes, and 500 cells were added to each well of a 6-well plate. Each group had 3 replicate wells. The medium was replaced every 48 hours, and the cells were fixed and stained after 14 days of incubation. Then, they were observed after washing with PBS.

### siRNA transfection

The cells were divided into the si-NC group, si-FKBP11-1 group, and si-FKBP11-2 group. Specific steps: MG63 cells and 143B cells were inoculated in a 12-well plate. When the cell density was approximately 40%, transient transfection was performed according to the above grouping concerning the instructions. The cells were transfected with DMEM and Lipofectamine transfection reagent, and the transfection efficiency was observed after 48 hours.

The siRNA primers in this paper were provided by GenePharma, and the sequences were as follows: Si-FKBP11-Homo334 (Si-FKBP11-1): sense (5′-3′): GCAGGUGAUUCCAGGUCUGTT, antisense (5′-3′): CAGACCUGGAAUCACCUGCTT;

Si-FKBP11-Homo581 (Si-FKBP11-2): sense (5′-3′): GGGUAUCACCUAUACAGAATT, antisense (5′-3′): UUCUGUAUAGGUGAUACCCTT.

### Cell proliferation assay

Monolayers of MG63 and 143B cells in the logarithmic growth phase were obtained from the adherent cells and inoculated in 96-well plates at a density of 1.0×10^3^/well. An appropriate amount of CCK-8 solution was added to each well, and the cells were allowed to react in the incubator for 2 hours. The absorbance value was measured with ELISA (detection wavelength 490 nm).

### Western blotting method

Proteins were extracted from osteosarcoma with RIPA lysates containing protein inhibitors. Quantify proteins with BCA. The proteins were denatured and subjected to protein gel electrophoresis. Then, the membrane was transferred and blocked with 5% skimmed milk powder for 1 hour. Diluted primary antibody was added and incubated for 4 hours at room temperature. The membrane was washed twice with PBST. Diluted secondary antibody was added and incubated for 1 hour at room temperature. The protein bands were analyzed with β-actin as the control.

### Apoptosis assay

Osteosarcoma cell apoptosis was measured according to the instructions of the Annexin V-FITC/PI Apoptosis Assay Kit (Catalog No. KGA107, Keygen, China). Then, 500 μl of buffer solution, 5 μl of Annexin V-FITC, and 5 μl of PI staining solution were added to 1 × 10^5^ cells/ml suspensions of osteosarcoma cells. The cells were mixed well and allowed to react in the dark for 20 min. Then, the cells were placed in a flow cytometer (Becton-Dickinson, USA) for apoptosis detection.

### Immunohistochemistry (IHC)

The samples of 15 cases of osteosarcoma were obtained from Nanchang First People’s Hospital. The tumors and para-cancerous tissues were excised during surgery. Osteosarcoma tissue specimens were soaked in wax, sliced into 4- to 6-μm slices, baked at 56°C for 2 h, and then dewaxed with xylene. Endogenous peroxidase was removed by a 3% hydrogen peroxide solution, and the antigen was recovered by CB antigen retrieval solution. (Nakazaki, Jinki, Oriental Gene, ZLI-9072). TBST containing 5% sheep serum (Nakasugi Golden Bridge, Origene, ZLI-9056) was blocked for 1 hour at room temperature, followed by incubation of FKBP11 primary antibody (protein tech AP6790a) drops on the sections overnight (1:50) in a 4°C refrigerator. The cells were incubated again the next day with anti-mouse/rabbit peroxidase secondary antibody (Bioss, 0294R) at 37°C for 2 h, followed by DAB (Solarbio, DA1010) color development.

### Statistical method

The gene expression profiles of the two groups of samples (normal bone tissue group and osteosarcoma group) were compared by the Wilcoxon signed-rank test. The reliability of the survival curves was verified by ROC curves, and the data were analyzed by GraphPad Prism 8.0 data software. Analysis of variance was performed by using a *t* test or one-way ANOVA. All cell culture experiments were performed in at least 3 independent experiments. Differences were considered statistically significant at *p* < 0.05.

## RESULTS

### Screening of tumor prognosis-related factors and construction of the risk assessment models

**Figure 1:**
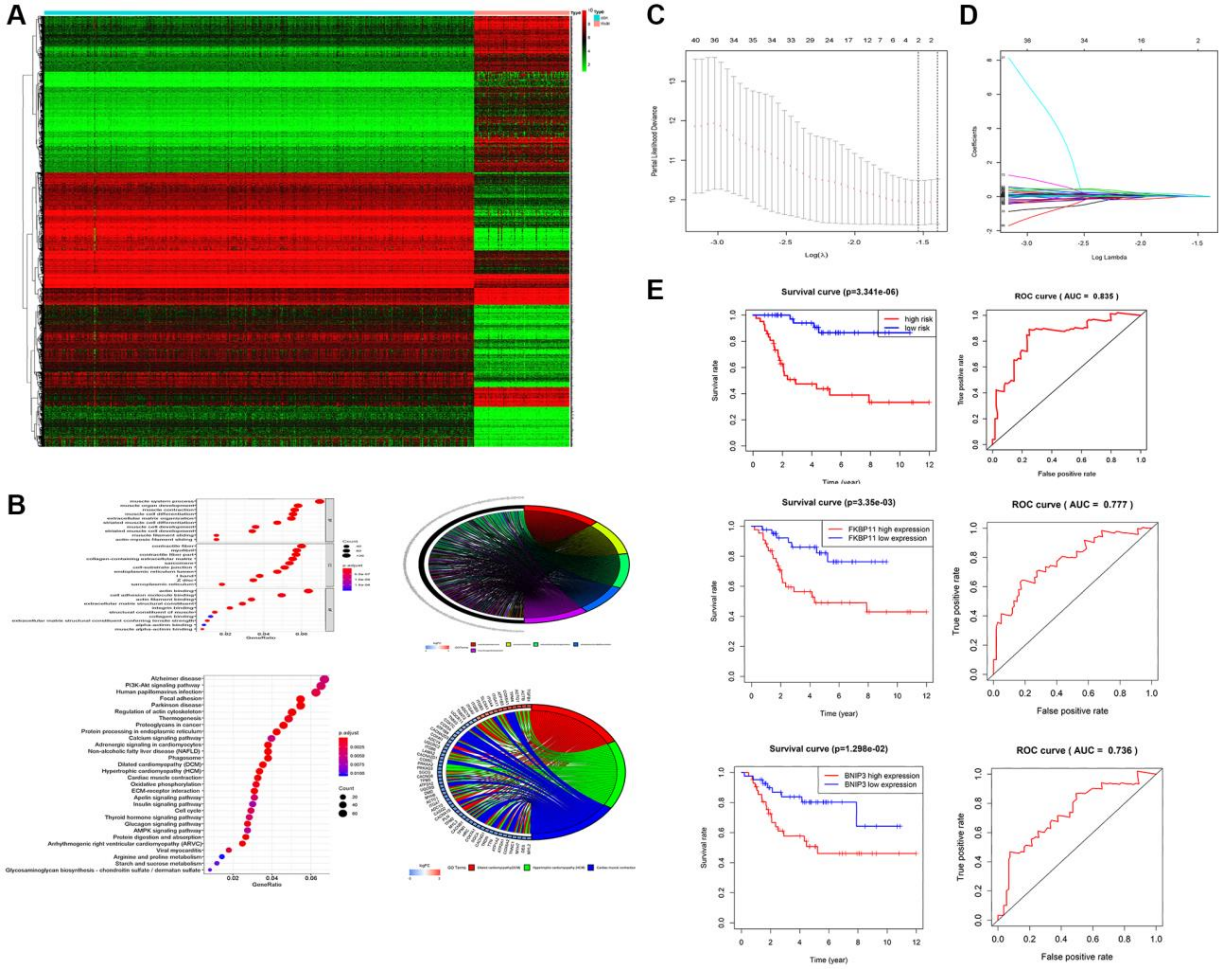
**Screening of tumor prognosis-related factors and construction of risk assessment models.** (**A**) Heatmap screening for differentially expressed genes. (**B**) Results of GO and KEGG enrichment analysis (*p* < 0.05). (**C**, **D**) Screening of tumor prognosis-related genes by LASSO regression analysis. (**E**) Reliability of risk regression models verified by survival and ROC curves of FKBP11 and BINP3. ^*^P < 0.05, ^**^P < 0.01, and ^***^p < 0.001.

To identify potential biomarkers in osteosarcoma, data including the mRNA expression profiles of the samples and relevant clinical information for the corresponding patients were collected from the GTEx and TARGET databases. The differentially expressed genes were screened, and a heatmap was used to present the differential expression (Figure 1A). In addition, GO and KEGG enrichment analyses were performed for these differentially expressed genes (Figure 1B). For the construction of the risk assessment model, univariate Cox regression was used to assess these differentially expressed genes. Some tumor prognosis-related genes were initially obtained according to the univariate Cox regression results (*p* < 0.05). Then, LASSO regression analysis (Figure 1C, 1D) and multivariate Cox regression analysis were performed on these genes that were initially screened, and two genes were found to be appropriate for building regression models: FKBP11 and BNIP3. The formula for the risk regression model was as follows: risk score = 0.724649371 × FKBP11 + 0.52423558 × BINP3. The risk assessment model formula was used to calculate the risk scores of these patients with osteosarcoma. Based on the risk scores obtained, all the abovementioned osteosarcoma patients were divided into high-risk groups and low-risk groups. Survival curves and ROC curves were drawn. The survival curves indicate better survival outcomes in the low-risk group than in the high-risk group. The area under the curve (AUC) was 0.835, suggesting that the risk assessment system has good prognostic predictive ability (Figure 1E). In addition, the survival and ROC curves of FKBP11 and BNIP3 further confirmed this result.

### The clinical value of a prognostic gene-based risk model for osteosarcoma

**Figure 2:**
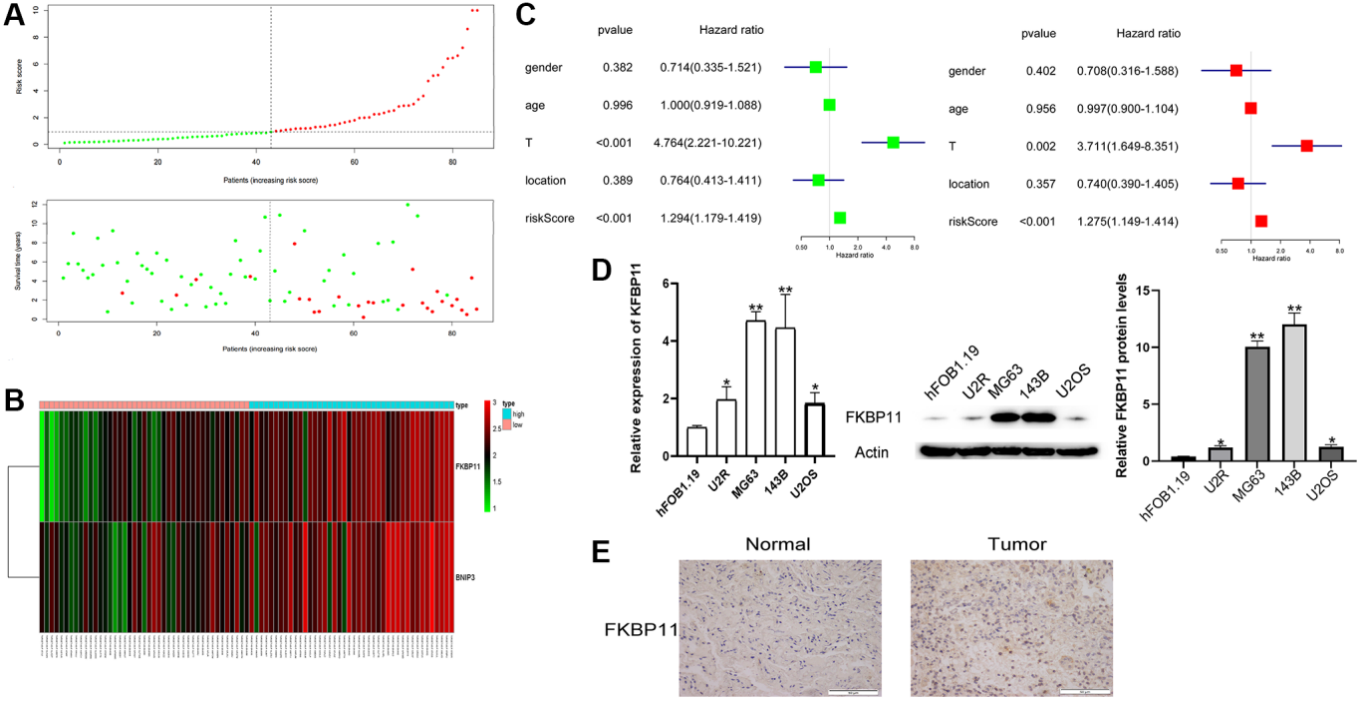
**The clinical value of a prognostic gene-based risk model for osteosarcoma.** (**A**) Patients’ risk scores were ranked from low to high, and scatter plots of risk scores and survival times were drawn. (**B**) Heatmap analysis of FKBP11 and BINP3 expression. (**C**) Univariate and multivariate regression analyses of factors correlated with osteosarcoma prognosis. (**D**) RNA was extracted from hFOB1.19 osteoblasts and osteosarcoma cells, and the expression of FKBP11 was measured by qRT-PCR. The protein expression of FKBP11 in hFOB1.19 and osteosarcoma cells was measured through western blotting. (**E**) Comparison of the expression of FKBP11 in osteosarcoma and paraneoplastic tissues. Scale bars represent 50 μm. ^*^P < 0.05, ^**^P < 0.01, and ^***^p < 0.001.

All patients’ risk scores were ranked from low to high by analyzing the sample risk score scatter plot and patient survival time graph. The higher the risk score was, the shorter the survival time (Figure 2A). The heatmap (Figure 2B) showed that the expression of FKBP11 and BNIP3 was significantly different in the high- and low-risk groups. Univariate and multivariate regression analyses of patient sex, patient age, tumor stage, tumor location, and risk score showed that tumor stage (*P* < 0.002) and risk score (*P* < 0.001) were significantly associated with the prognosis of patients with osteosarcoma (Figure 2C). We then chose FKBP11 for further functional study. To further analyze the expression of FKBP11 in osteosarcoma, we compared the expression of FKBP11 in HFOB1.19 cells and osteosarcoma cells by using qRT-PCR and western blotting. The results clearly showed that the mRNA and protein levels of FKBP11 were higher in osteosarcoma cells than in HFOB1.19 osteoblasts (Figure 2D). We further assessed the expression of FKBP11 in osteosarcoma tissues. The immunohistochemistry results showed that the expression of FKBP11 in osteosarcoma tissues was higher than that in para-cancerous tissues (Figure 2E).

### FKBP11 suppresses osteosarcoma cell proliferation, invasion and migration

**Figure 3:**
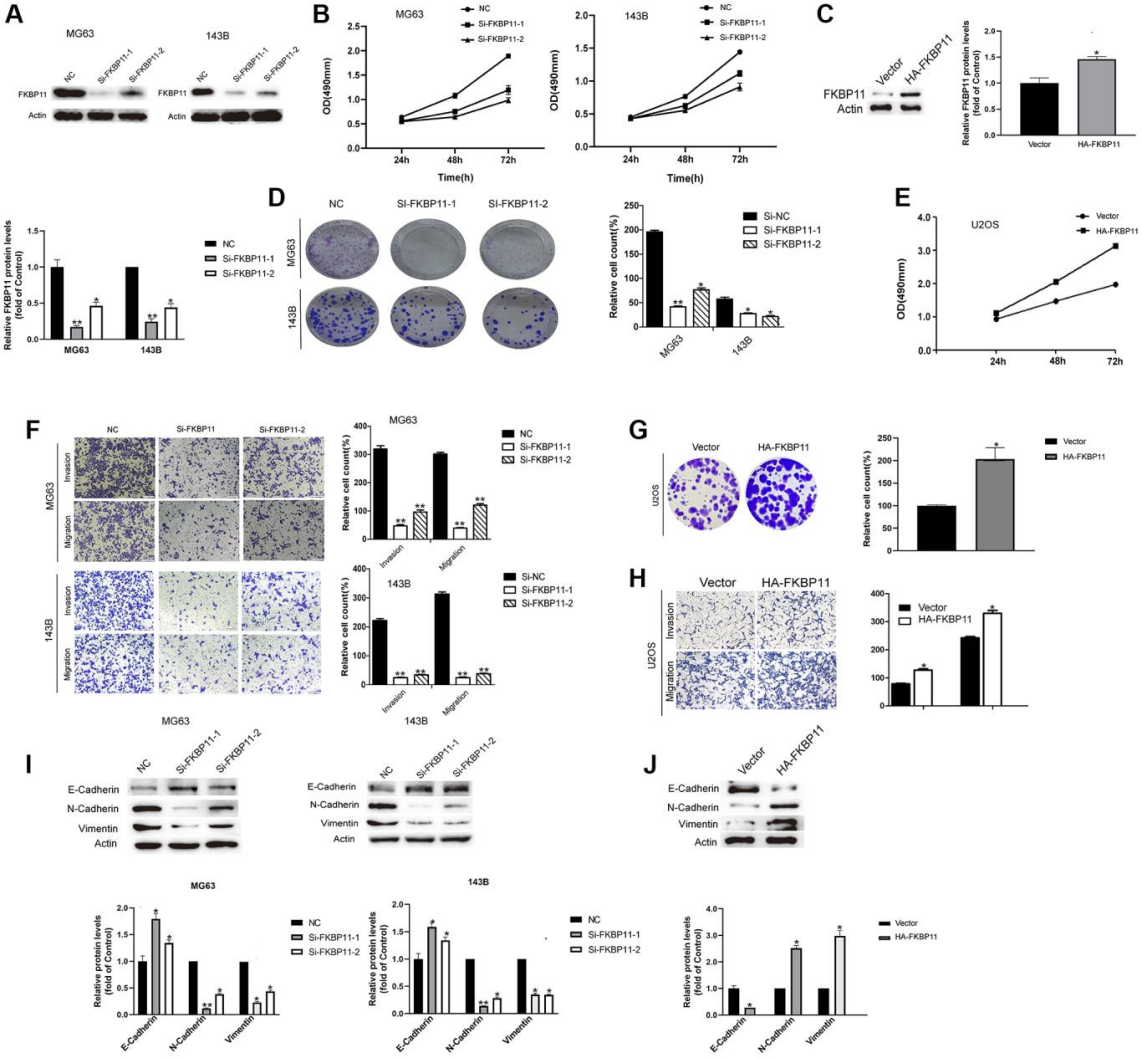
**FKBP11 suppresses the proliferation, invasion and migration of osteosarcoma cells.** (**A**) The knockdown efficiency of FKBP11 in osteosarcoma cells was measured through qRT-PCR and western blotting after the transfection of MG63 and 143B cells with Si-NC and Si-FKBP11, respectively, for 48 h. (**B**) The proliferative capacity of osteosarcoma was measured by CCK-8 assay. (**C**) FKBP11 overexpression was detected by western blotting. (**D**) Results of colony formation assays for osteosarcoma cells. (**E**) The proliferative capacity of osteosarcoma cells was measured by CCK-8 assay. (**F**–**H**) Results of invasion and migration assays of osteosarcoma cells. Scale bars represent 100 μm. (**I**–**J**) The protein levels of E-cadherin, N-cadherin, and Vimentin were measured through western blotting analysis. Data are presented as the means ± SDs (*n* = 3). Comparison of Si-FKBP11 and NC, ^*^P < 0.05, ^**^P < 0.01, and ^***^p < 0.001.

We examined the functional roles of FKBP11 in osteosarcoma. FKBP11 was knocked down by using siRNAs in MG63 and 143B cells, and knockdown was confirmed by western blotting (Figure 3A). Then, we assessed proliferation, invasion, migration, and colony formation in osteosarcoma cells upon FKBP11 silencing. After silencing FKBP11 in MG63 and 143B cells, the proliferation of osteosarcoma cells was significantly suppressed according to the results of the CCK-8 assay (Figure 3B). Colony formation was also suppressed after silencing FKBP11 (Figure 3D). In addition, when FKBP11 was silenced in MG63 and 143B cells, osteosarcoma cells had decreased migratory and invasive abilities (Figure 3F). Accordingly, silencing FKBP11 increased the protein level of the epithelial marker E-cadherin and reduced the protein levels of the mesenchymal markers N-cadherin and vimentin (Figure 3I). To explore whether the overexpression of FKBP11 might have the opposite effect to that of the knockdown of FKBP11. FKBP11 was overexpressed in U2OS cells (Figure 3C). As shown in Figure 3E, 3G, 3H, and 3J, overexpression of FKBP11 induced effects opposite to those induced by FKBP11 knockdown.

### FKBP11 significantly increases the apoptosis rate in osteosarcoma cells and affects the MAPK pathway

**Figure 4:**
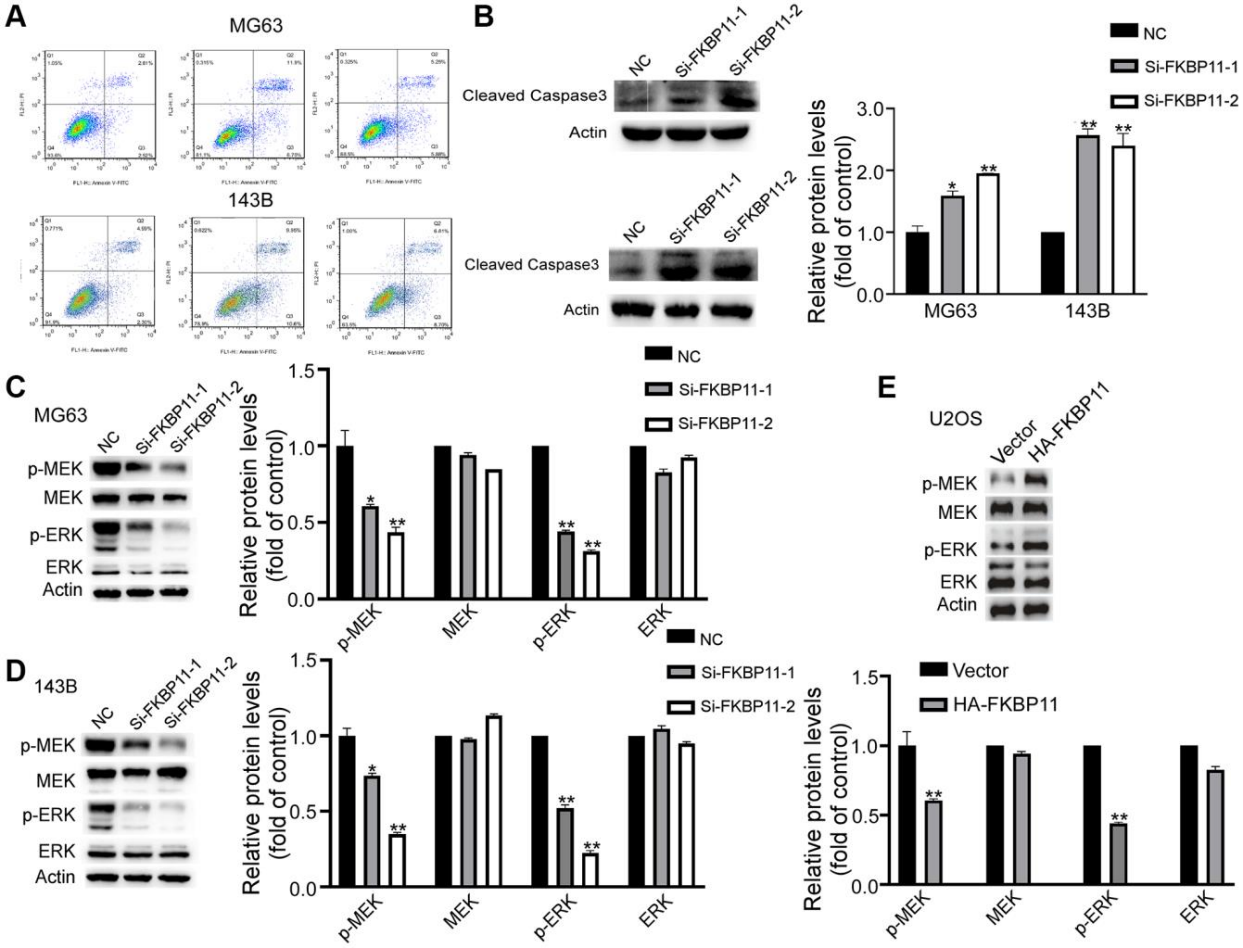
**FKBP11 significantly increases the apoptosis in osteosarcoma cells and affects the MAPK pathway.** (**A**) Apoptosis was measured with an apoptosis kit. (**B**) Protein expression of cleaved caspase-3 was measured through western blotting after transfection of MG63 and 143B cells with Si-NC and Si-FKBP11 for 48 h. (**C**–**E**) The protein expression of p-MEK, MEK, p-ERK, and ERK in MG63 and 143B cells was measured by western blotting. ^*^P < 0.05, ^**^P < 0.01, and ^***^p < 0.001.

Next, we further examined the effect of FKBP11 in regulating osteosarcoma cell apoptosis. The results showed that after knocking down FKBP11, the apoptosis rate of cells was significantly increased compared with that of control cells (Figure 4A). Consistently, the expression of the apoptotic marker caspase 3 markedly increased (Figure 4B). It was reported previously that the FK506 binding protein-mediated MAPK pathway can alleviate prion-induced neurodegeneration [12]. The role of the MAPK pathway regulated by FKBP52 in polycystic ovary syndrome (PCOS) has also been reported [13]. We then explored whether FKBP11 plays a tumor-promoting role in osteosarcoma through the MAPK pathway. Compared si-NC (control) transfection, siFKBP11 transfection suppressed the phosphorylation of MEK/ERK (Figure 4C, 4D), while overexpression of FKBP11 promoted the phosphorylation of MEK/ERK (Figure 4E). These results suggest that FKBP11 may have an important impact on the progression of osteosarcoma via the MAPK signaling pathway.

## DISCUSSION

A large number of genes showed greater than 4-fold increases in expression between osteosarcoma tissues and normal tissues. GO and KEGG enrichment analyses were performed on these genes. This analysis revealed that FKBP11 controls the transcription of multiple invasion- and metastasis-related genes, such as integrins, matrix metalloproteinases (MMPs), and A Disintegrin and Metalloproteinase (ADAM) proteins, as well as the TGF signaling pathway, which is the most differentially enriched pathway between osteosarcoma tissues and normal tissues at the transcriptional level. GO enrichment analysis revealed that the differentially expressed genes were mainly enriched in muscle system processes, muscle contraction, extracellular matrix organization, rhabdomyocyte differentiation, and muscle organ development. KEGG enrichment analyses revealed that the differentially expressed genes were mainly enriched in dilated cardiomyopathy, hypertrophic cardiomyopathy, and myocardial contraction. Two genes, FKBP11 and BINP3, were finally screened by univariate Cox analysis, LASSO regression analysis, and multivariate Cox regression analysis. Then, we constructed a risk assessment model, which was used to draw a scatter plot of survival time for the whole cohort. Finally, we performed univariate and multifactorial analyses to determine the association of patient sex, patient age, tumor stage, tumor location, and risk model score with tumor prognosis; tumor stage and risk model score were statistically significant prognostic factors. This further suggests that this risk assessment model can predict the prognosis of patients with osteosarcoma.

FKBP11 (FK506-binding protein) belongs to the FK506-binding protein family, which binds immunosuppressive drugs (including FK506 and rapamycin) [14]. The FKBP11 gene encodes a 22 kDa preprotein containing an amino acid N-terminal sequence consisting of 25 residues. Cleavage of the head peptide leaves a 19 kDa protein, so FKBP11 was also named FKBP19 [15]. FKBP11 mRNA is abundant in secretory tissues such as the pancreas, stomach, and salivary glands [14]. FKBP11 expression progressively increases during the development of hepatocellular carcinoma (HCC). The results suggested that FKBP11 may be a potential early marker of HCC; the expression of FKBP11 is also increased in melanoma, but the exact mechanism of action is unclear [16]. Furthermore, some researchers [17] studied the promoter of FKBP11 and identified a conserved nucleotide stretch that is similar to the unfolded protein response element (UPRE). It is the classical binding site for XBP1.

It has been reported that the MAPK pathway contains the following main components: MAPKK kinase (MAPKKK), MAPK kinase (MAPKK), and MAPK [11]. Extracellular signal-regulated kinase (ERK) and MEK are important members of the MAPK and MAPKK families [18]. Xu holds the view that microRNA-497 can inhibit the development of osteosarcoma via the MAPK/Erk pathway [19]. It has also been reported that MEK inhibition can inhibit osteosarcoma cell growth and thus reduce tumor growth *in vivo*. According to the study, the growth of osteosarcoma cells was diminished when MEK was inhibited, thus reducing tumor growth in the body [20]. These studies all suggest that the MAPK pathway exerts a weighty influence on the process of osteosarcoma development.

Our experiments validated the role of FKBP11 in the development of osteosarcoma at the tissue and cellular levels. In our study, we confirmed that the development of osteosarcoma is regulated by the prognostic factor FKBP11. Moreover, we found that the knockdown of FKBP11 inhibited the MAPK signaling pathway, suggesting that FKBP11 may promote the development of osteosarcoma via the MAPK pathway. This study provides a better understanding of the pathogenesis of osteosarcoma. In conclusion, the potential prognostic factor associated with patient survival, FKBP11, can predict the prognosis of osteosarcoma patients based on its effect on the development of osteosarcoma. These findings lay the groundwork for future research into the role of FKBP11 in osteosarcoma treatment. The utility of the prognostic factor FKBP11 remains to be further explored in animal and clinical trials.
